# Comment on Barczyk et al. Potential Clinical Applications of Ozone Therapy in Dental Specialties—A Literature Review, Supported by Own Observations. *Int. J. Environ. Res. Public Health* 2023, *20*, 2048

**DOI:** 10.3390/ijerph20247154

**Published:** 2023-12-08

**Authors:** Gerardo Tricarico, Valter Travagli

**Affiliations:** 1 Presidio Ospedaliero Sant’Andrea, 13100 Vercelli, Italy; 2 Dipartimento di Biotecnologie, Chimica e Farmacia, Università degli Studi di Siena, Viale Aldo Moro, 2, 53100 Siena, Italy

A review from Barczyk et al. has just appeared online [1]. This topic is relevant and of such interest that we take the liberty of pointing out a recent publication, which is not listed among the references given and whose objective is to improve the knowledge of the qualitative/quantitative characteristics of ozone and its derivatives [2]. Unfortunately, however, there are inaccuracies right from the introduction, including with regard to the ozone concentrations and their methods of use.

The therapeutic procedure using ozone and its derivatives needs more visibility, standardisation and accepted criteria for documentation in order to move out of the realm of complementary medicine and take its rightful place as a part of the therapeutic armamentarium available to dentists. To this end, we take the liberty of suggesting the publication of an appropriate corrigendum by the authors, or the composition of an otherwise interesting, though not entirely innovative, review.
